# Feasibility of a prospective, longitudinal study of resilience among young military recruits with an embedded laboratory sub-study: the ARMOR pilot trial

**DOI:** 10.1186/s40814-024-01573-6

**Published:** 2025-03-08

**Authors:** Andrea C. Hitz, Michelle Hubbling, Annika Hodges, Emily Hagel Campbell, Ann Bangerter, Melissa A. Polusny

**Affiliations:** 1 Minneapolis VA Health Care System, Minneapolis, MN USA; 2 Department of Psychiatry and Behavioral Sciences, University of Minnesota Medical School, Minneapolis, USA; 3 Center for Care Delivery Outcomes Research, Minneapolis, MN USA

## Abstract

**Background:**

Despite extensive resilience research with military personnel, progress in developing effective resilience-specific interventions for military service members (MSM) has been limited. To inform the design of effective prevention and intervention strategies, a better understanding of the neurocognitive, behavioral, and social processes associated with resilience is needed. This paper reports on a pilot study testing the feasibility of methods and procedures, to be used in the Advancing Research on Mechanisms of Resilience (ARMOR) longitudinal cohort study. Pilot trial objectives were to evaluate the feasibility of recruitment, retention, and data collection against pre-specified progression criteria for determining whether to proceed with the large-scale study.

**Methods:**

This pilot trial used a pre-test/post-test design with an embedded laboratory sub-study. Participants were young recruits who had recently enlisted in the US Army National Guard and had not yet shipped to Basic Combat Training (BCT). Recruitment and baseline data collection at local armories involved computerized self-report measures and neurocognitive tests. Participants completed a web-based follow-up survey on personal devices after BCT. A subset of participants was recruited to complete laboratory procedures pre- and post-BCT, including clinical interviews and neurobehavioral tasks. To evaluate the feasibility of collecting real-time assessments of military stressors, participants were randomized to receive web-based surveys during BCT.

**Results:**

Among the 105 military service members approached, 101 (96.2%) were recruited. Baseline data collection and retention methods were considered feasible; the response rate to the follow-up survey was over 70%. Recruitment and data collection for the laboratory sub-study were also considered feasible; more than 90% of participants completed follow-up laboratory visits. The collection of web-based surveys during BCT and computerized neurocognitive testing at follow-up was not considered feasible; the large-scale study will remove these components.

**Conclusions:**

Progression to the large-scale trial, with design refinements, was concluded. Lessons learned and recommendations for future research are discussed.

## Key messages regarding feasibility

- *What were the uncertainties regarding feasibility?* The uncertainties regarding feasibility were the ability to recruit, retain, and collect data, particularly computerized cognitive and intensive laboratory data, from young military recruits.
- *What are the key feasibility findings?* All predetermined progression criteria were met, and the overall trial design and study methods proved to be feasible.
- *What are the implications of the feasibility findings for the design of the main study?* The findings indicate that a full-scale longitudinal study with an embedded laboratory sub-study is feasible; however, collecting web-based survey data from participants while they are at BCT and collecting computerized cognitive measures at follow-up using personal devices are not feasible.

## Introduction

### Background and rationale

The occupation of warfighting is inherently associated with a variety of stressors and serious hazards that can adversely impact the health, well-being, and performance of its members, making military service an ideal context for studying resilience. Despite the importance of resilience to the military, progress in the development of effective resilience-specific interventions for military service members (MSM) has been limited [4]. Numerous longitudinal studies have mapped distinct patterns (i.e., trajectories) of posttraumatic stress symptoms among MSM following combat deployment. While these studies have shown that a resilient trajectory is the most common response following deployment [2, 14, 17], few prospective studies have investigated trajectories of positive adaptation among young recruits beginning early in their military career [22]. Young military recruits who have recently enlisted and not yet shipped to Basic Combat Training (BCT) provide an especially important population for studying resilience because they have not yet been exposed to military-related stressors, and findings could help guide the development of interventions for this population [12]. In addition, few studies have investigated the mechanisms associated with resilience. Most existing studies of resilience in military populations have relied on self-report measures. Although invaluable, reliance on self-report measurement alone is associated with several limitations, including the potential for systematic nonresponse bias and monomethod bias [1, 5, 23]. Prospective, longitudinal studies of resilience are beginning to incorporate multilevel approaches [3, 21], including neuroimaging data [19]. However, very few have focused on young military recruits at the onset of their careers [24].

To inform the design of effective resilience-enhancing strategies for MSM, we planned a large-scale prospective, longitudinal cohort study with an embedded laboratory sub-study. For the large-scale study, a cohort of military recruits who recently joined the US Army National Guard will be recruited, baseline surveys will be collected from them at military installations before they enter BCT, and follow-up surveys will be collected from the cohort at multiple time points after they return from BCT. The main study also incorporates an embedded laboratory sub-study involving clinical diagnostic interviews, neurocognitive testing, and DNA sampling. Full details can be found in the trial protocol [16]. Because large, prospective, longitudinal studies are costly and resource-intensive endeavors, it was necessary to first assess the feasibility of methods and procedures to be used in the larger study [25].

While our team has a long history of successfully conducting longitudinal studies with National Guard soldiers in the context of military deployment [6, 7, 13, 14], we were uncertain regarding the feasibility of recruiting and retaining a cohort of younger military recruits, collecting data in the field via electronic surveys, and incorporating an embedded laboratory sub-study. Studies of health and well-being in emerging and young adult populations suggest that these age groups may be especially difficult to recruit for longitudinal studies [9]. Previous research has also shown that attrition from longitudinal studies is highest among younger adults, especially those with less education [26]. With respect to the feasibility of data collection in the field, researchers have shifted away from using well-established mailed survey methods and increasingly adopted electronic survey methods [10]. Electronic surveys offer benefits such as convenience, accessibility, and reduced staffing needs, as well as the ability to incorporate performance-based neurocognitive measures. For example, the Penn Computerized Neurocognitive Battery (CNB) now enables researchers to integrate neurocognitive measures into large studies [11]. However, the administration of electronic surveys requires that participants have access to electronic devices and reliable Internet access, and these resources may not be readily available when data are collected in the field (e.g., at military installations during drill training). In addition, the Penn CNB was not designed for remote administration on personal devices [8], and test developers recommended that a proctor assist with test administration to address any issues participants might encounter. While we planned to address these potential challenges in the large-scale study by supplying study laptops for baseline data collection, we were uncertain of whether it was logistically feasible to collect electronic surveys, including Penn CNB measures, from large groups of soldiers in remote settings. To minimize biases from retrospective self-reports, resilience studies should collect data on participants’ stressor exposure as close to the actual events as possible. In planning the larger study, we considered the possibility of assessing soldiers’ exposure to stressors *during* BCT. Few studies by civilian researchers have collected data from military personnel during deployment or training [7], making the feasibility of collecting brief web-based survey data during BCT uncertain. Finally, laboratory methods provide valuable information for evaluating potential mechanisms of resilience but place a high burden on participants and can be costly. The feasibility of recruiting participants to take part in the laboratory sub-study as well as collecting complete data in a timely manner was also unclear.

Therefore, the aims of this study were to assess the feasibility of recruiting, retaining, and collecting data from young military recruits.

## Methods

### Aim and design

This pilot study was designed as a small-scale test of the methods and procedures to be used in the larger Advancing Research on Mechanisms of Resilience (ARMOR) prospective, longitudinal cohort study. In the planned large-scale study, resilience will be operationalized as a trajectory of positive adaptation (across domains of social-occupational functioning, internalizing symptoms, and externalizing problems) in response to BCT. Study measures were chosen based on our goal of measuring resilience as a dynamic process that unfolds over time [12]. The goal of this pilot study was not to test hypotheses about the effects of BCT on soldiers’ resilience but, rather, to assess the feasibility of participant recruitment, retention, and data collection procedures for use in the large-scale study. Therefore, our measurement outcomes were identical to the planned large-scale study. For more in-depth detail of selection/description of relevant outcome measures, see Polusny et al. [15].

**Fig. 1:**
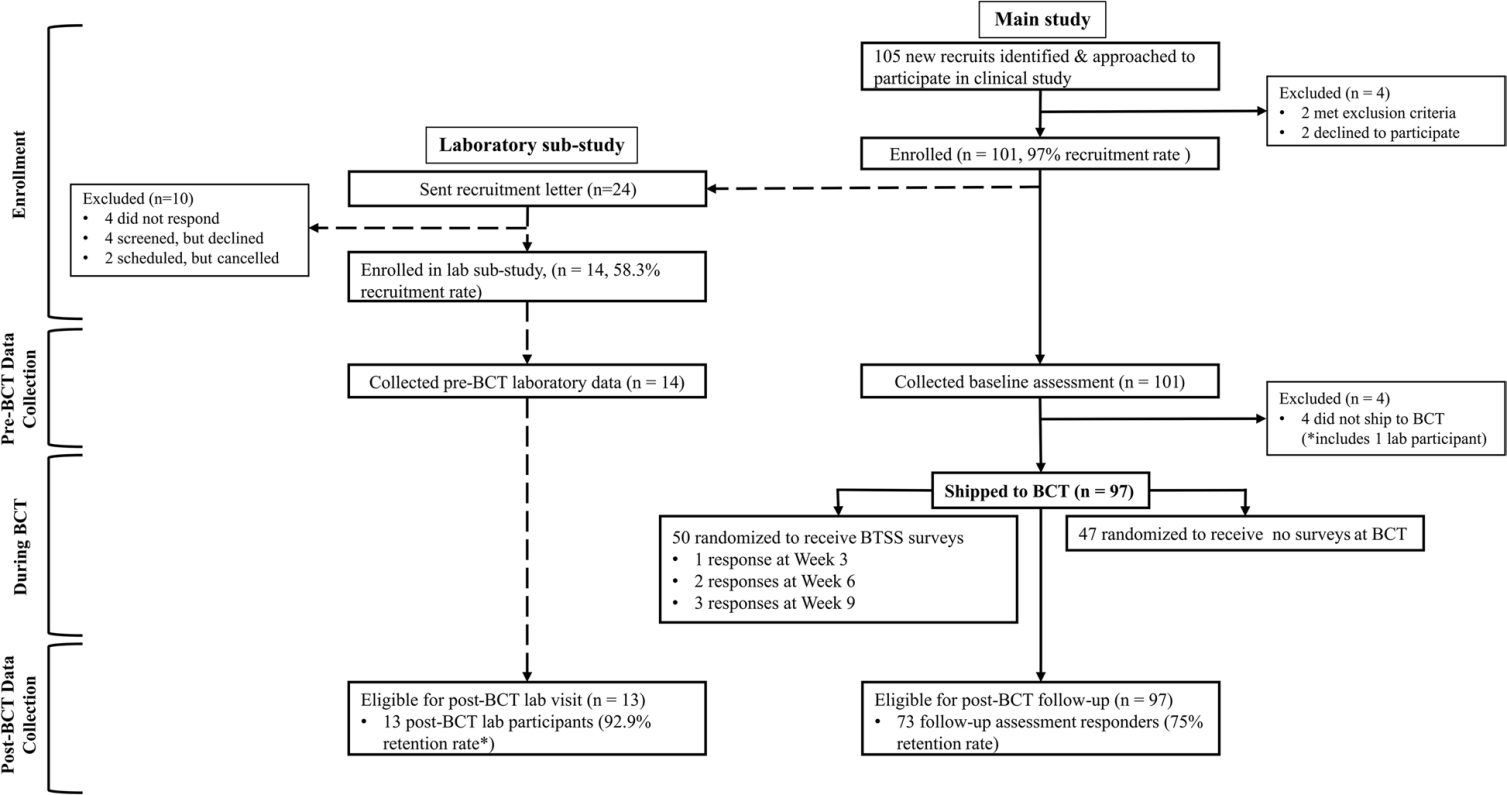
Flow of participants through the pilot study

As shown in Fig. 1, this pilot study employed a pre-test/post-test design with an embedded laboratory sub-study and a nested randomized controlled trial (RCT). Specifically, the pre-test/post-test design allowed us to test the logistical feasibility of collecting web-based surveys incorporating performance-based neurocognitive testing via study laptops at military installations as well as the feasibility of collecting these measures after BCT via participants’ personal devices. The embedded laboratory sub-study allowed us to test the feasibility of recruiting and retaining young military participants to participate in time-intensive, in-person study visits during times of transition (prior to BCT and after returning from BCT). Finally, we employed a nested RCT to explore the feasibility of collecting stressor exposure data via brief web-based surveys while participants were stationed at BCT.

The purpose of this paper is to report on the feasibility of the research protocol for the large-scale ARMOR study. Our objectives were to determine the following: (1) the feasibility of participant recruitment methods, (2) the feasibility of study retention methods, and (3) the feasibility of data collection procedures.

### Ethics

This study was approved by the University of Minnesota and Minneapolis VA Health Care System Institutional Review Boards. The Army Human Research Protection Office reviewed the study and determined that all applicable federal, Department of Defense (DOD), and Army regulations and directives were met. Research procedures were conducted by a civilian research team with no affiliation to the National Guard.

### Participants and procedures

As a detailed description of the pilot study methods is presented elsewhere [15], we present a brief summary below. Participants were recently enlisted in the US Army National Guard, aged 18 or older, and scheduled to ship and return from BCT within the 12-month study period. Individuals previously exposed to BCT (i.e., as part of previous military service) were excluded. Because the laboratory sub-study was considered greater than minimal risk and minors are prohibited from assenting for such studies [18], we excluded minors to allow us to complete feasibility testing within the 12-month pilot phase. During the pilot phase, we sought IRB approval for a waiver of parental consent to allow 17-year-old soldiers to participate in the survey component during drill training. This protocol amendment was approved by IRB after the pilot trial recruitment period ended.

Figure 1 displays the flow of participants through the progression of the pilot trial. Participant recruitment and baseline data collection occurred at local Army National Guard armories before the recruits shipped to BCT. During drill training, investigators conducted briefing sessions with groups of recruits to explain the purpose, nature, and risks of the study. The voluntary and confidential nature of the study was emphasized. Military command was not present during briefings to increase participant confidentiality. Participants were informed that they would receive an invite to complete a self-administered electronic survey after returning from BCT as well as potentially several invites during BCT. Extensive contact information was collected to facilitate tracking participants longitudinally.

A subsample of participants enrolled in the pilot cohort was recruited to complete the laboratory sub-study (see dashed lines in Fig. 1). Recruitment for the laboratory component was initiated by invitation letters sent to participants with anticipated BCT ship dates more than 2 weeks after baseline data collection, and follow-up calls were made to screen participants for safety criteria for an MRI scan (e.g., no metal implants) and schedule lab visits. Lab participants were enrolled sequentially until our recruitment goal was reached.

Data were stored in password-protected folders on a secure share drive that was accessible only to authorized study personnel. The participants were assigned study ID numbers that were used for data transfer, communication, and analysis purposes. Any hard copies of the data collection forms were stored in locked file cabinets, in a locked storage room, and in a secured building.

#### Survey procedures

Baseline questionnaires were administered at armories via a confidential and secure link to Qualtrics using military Wi-Fi and study Chromebooks. Select tests from the Penn CNB [11] were administered at baseline via the Penn CNB website. Because we aimed to test the feasibility of the methods and procedures used in the large-scale ARMOR study, the battery of assessments administered in the pilot was identical to those planned for the large-scale study (see Polusny et al. [16] for details). Baseline data collection took approximately 75 min. Military regulations dictated that participants could not be compensated by the study for data collection during drill weekends. Following baseline data collection, participants were sent a thank you card and military challenge coin, and contact information provided by participants was verified and confirmed. After returning from BCT, the participants were contacted by email and sent a confidential and secure link to complete a follow-up survey via Qualtrics using a personal device. The same set of self-report measures administered at baseline were given in the follow-up survey. Participants were provided detailed instructions on how to complete the neurocognitive tests via the Penn CNB website on their personal device. To optimize follow-up survey completion, survey non-responders received up to four emails and three calls to remind them to complete the follow-up survey.

#### Laboratory sub-study procedures

Pre-BCT lab visits were conducted at the Minneapolis VA Medical Center and University of Minnesota. The participants completed a clinical interview and short battery of self-report measures (1–2 h), provided a DNA sample via a blood draw, and completed a series of performance-based tasks (2.5–3 h) involving electroencephalography (EEG) at the Minneapolis VA Medical Center. At the pre-BCT lab visit only, participants also completed a functional magnetic resonance imaging (fMRI) assessment (2.5–3 h) at the University of Minnesota’s Center for Magnetic Resonance Research (CMRR). After returning from BCT, participants completed the same procedures, excluding fMRI, at a post-BCT lab visit. Participants were compensated US $100 for completing the pre-BCT lab visit at the Minneapolis VA, US $100 for completing the pre-BCT lab visit at CMRR, and US $200 for completing the post-BCT lab visit at the Minneapolis VA.

#### Additional procedures

To explore whether collection of data assessing soldiers’ exposure to stressors during BCT was feasible, we conducted a small, randomized controlled trial (RCT) nested within the larger study design. Randomization was assigned by study ID at the time of enrollment using a computer-generated randomization with a simple blocking procedure. A total of 50 participants were set to receive brief web-based surveys at three time points during BCT (weeks 3, 6, and 9). The remaining participants did not receive any survey invites during BCT. Each survey included the 14-item Basic Training Stressor Scale (BTSS; [15]) which was estimated to take less than 5 min to complete. Participants were sent an email containing a confidential and secure survey link that was open for 7 days; nonresponders received two reminder emails.

Administrative data linked to all soldiers approached for the study was obtained from the Minnesota Army National Guard command. This information included demographics, anticipated BCT ship and return dates, and Armed Forces Qualification Test (AFQT) scores. Demographics and AFQT scores were used to examine representative of the enrolled cohort. Training dates were used to target laboratory recruitment efforts at participants with ship dates later than 2 weeks post-enrollment and to determine follow-up outreach timelines.

### Outcomes

Our primary feasibility outcomes were recruitment and retention rates. For the survey component, recruitment was defined as the number of soldiers enrolled per the number approached at recruitment briefings. For the laboratory component, recruitment was defined as the number of cohort participants who consented to participate in the laboratory sub-study per number approached. Retention (survey component) was defined as the number of participants who responded to the follow-up survey per the total number eligible for follow-up. Retention in the laboratory component was defined as the number of participants who completed post-BCT lab visits per the number eligible for follow-up. Our secondary outcomes were representativeness of the pilot sample (i.e., enrollment of at least 25 women) and data completion (i.e., proportion of participants who provided complete surveys or completed all lab tasks).

During the planning phase of our UG3 Exploratory/Developmental Phased Award Cooperative Agreement with the National Institutes of Health’s National Center for Complementary and Integrative Health (NCCIH), we worked with NCCIH and our stakeholders (local National Guard command) to establish predefined quantitative progression criteria to determine whether to proceed with the large-scale trial. Table 1. summarizes the pre-determined progression criteria for each of our feasibility outcomes.

**Table 1:** Summary of pilot trial objectives, progression criteria, and outcomes

Objectives	Criteria for demonstrating feasibility	Evaluation outcome
1. Demonstrate ability to recruit participants for the study	a) Achieve target enrollment of ≥ 100 participants (minimum of 25 females) for inclusion in the pilot survey study. Recruitment will require approaching < 133 service members (i.e., > 75% recruitment rate) within a 4-month recruitment period	Achieved
	b) Recruit at least 14 sample participants for inclusion in the laboratory sub-study. Recruitment will require approaching < 35 service members (i.e., > 40% recruitment rate)	Achieved
2. Demonstrate ability to retain participants in the study	a) Achieve post-BCT survey response rate (retention rate) of ≥ 65%	Achieved, some study modifications planned
	b) Achieve post-BCT laboratory visit retention rate of ≥ 85%	Achieved
3. Demonstrate the ability to collect complete study data	a) Achieve ≥ 90% complete surveys among survey responders	Partially achieved, some study modifications planned
	b) Achieve ≥ 80% completion of all lab tasks	Achieved
**Exploratory aim**	**Evaluation plan**	**Outcome**
Explore ability to collect brief surveys during BCT	Assessment of whether it is feasible to collect BCT stressor data in real time during BCT or post-BCT only	Soldiers were unable to respond during BCT

#### Feasibility of study recruitment methods

To evaluate our ability to recruit participants for the clinical study, we examined the recruitment rates for the survey cohort and laboratory sub-study cohort. The progression criteria for the survey component included the following: enrollment of at least 100 participants, including 25 females, within the 4-month study recruitment timeline, and a recruitment rate of at least 75%. Progression criteria for the laboratory sub-study included the following: recruitment of a minimum of 14 participants to complete lab visits and a recruitment rate of at least 40%.

#### Feasibility of study retention methods

To evaluate our ability to retain participants in the study, we examined the retention rate for the follow-up survey and the laboratory sub-study. We set the goal of achieving at least a 65% response rate for the follow-up survey. For the laboratory sub-study, we set the goal of achieving an 85% retention rate. We also had a secondary objective for testing the feasibility of retention methods. We wanted to determine the best timeline for initiating follow-up outreach as well as length of time or window needed for survey completion to optimize retention.

#### Feasibility of data collection methods

To evaluate our ability to collect study data, we determined the proportion of the cohort who provided complete surveys and the proportion of lab participants who completed all lab tasks. A complete survey was defined as having less than 10% missing data on key study variables. We set the goal of collecting complete surveys at each time point from 90% or more of survey responders. Lab tasks to be completed during a laboratory visit included completion of the clinical interview, self-report measures, four EEG tasks, and at the pre-BCT visit only, the MRI session. We set a goal of achieving a minimum of 80% or greater completion of all lab tasks at each time point.

#### Exploratory aim

To explore the feasibility of collecting brief surveys during BCT, we examined response rates to web-based surveys administered at weeks 3, 6, and 9 of BCT. No progression criteria were defined for this exploratory outcome.

#### Protocol modifications

We closely monitored feasibility outcomes in real time throughout the pilot trial, identified challenges and troubleshooted with NCCIH and our military stakeholders as needed, and carefully documented all implementation challenges and solutions. The following minor protocol modifications were made during the study. First, we varied the timing of when follow-up outreach procedures were initiated with participants relative to their scheduled BCT return dates. We sought to collect follow-up data, including assessment of BCT stressor exposure, as close in time as possible to actual BCT events. Therefore, we initially began follow-up outreach procedures on the day the soldier was scheduled to return from BCT but observed lower than expected response responses. As we interacted with participants during the follow-up period, we learned that BCT return dates in our records were approximate, and that some of our initial outreach had occurred before participants returned from BCT. To account for this, we delayed the start of the follow-up outreach process to begin 2 weeks after estimated BCT return and observed that follow-up response rates improved.

Second, we altered how we implemented the follow-up Penn CNB protocol (neurocognitive testing completed through the Penn CNB website on participants’ personal devices). Initially, the follow-up email invitation included detailed instructions for accessing the Penn CNB website (e.g., ensuring their personal devices had compatible browsers) and the confidential link to the follow-up Qualtrics survey. When the Penn CNB was linked with the follow-up Qualtrics survey, we observed lower than expected follow-up response rates. These observations prompted us to adjust the protocol such that when participants finished the follow-up Qualtrics survey, an email was triggered that included the Penn CNB instructions and link. By administering the follow-up survey and Penn CNB separately, we were able to disentangle the follow-up survey response rate from that of the Penn CNB. While follow-up response rates improved, responses to the Penn CNB remained low.

Finally, we made modifications to the protocol for implementing web-based survey procedures during BCT. Originally, for each survey (surveys were sent weeks 3, 6, and 9 of BCT), an email was sent alerting the participant that a survey link would be coming soon, a second email was sent containing the survey link, and three additional reminder emails were sent to nonresponders (up to five emails per survey). However, extremely low response rates and feedback that the quantity of emails was overwhelming led us to decrease our emails and abandon the trial (see response rate details in the “Results”).

### Sample size and data analysis

Given that the goal of this pilot study was not to test hypotheses, power calculations for the proposed pilot sample size were not performed. Instead, pilot sample size was based on primary feasibility objectives (i.e., the number of participants needed to reasonably evaluate progression criteria related to rates of recruitment and retention), practical considerations including participant flow (e.g., availability of soldiers preparing to ship to BCT), and budgetary constraints (e.g., cost of laboratory procedures such as fMRI). With a target sample of 100 for the survey study, we can estimate feasibility parameters as follows: enrollment rate of 75% (100/133) with a 95% CI from 68 to 82% and post-BCT survey response rate of 65% (65/100) with a 95% CI from 56 to 74% [25]. This target sample size was deemed reasonable by our military stakeholders, the local National Guard, and our NCCIH funding partner. The sample for the laboratory sub-study was originally set at 10 (10% of the full pilot cohort sample). This was chosen to test the large-scale study goal of recruiting 10% of the full cohort into the laboratory sub-study cohort. However, NCCIH expressed concern that 10 participants would be insufficient to demonstrate recruitment and retention feasibility, so we increased the pilot lab sample size to 14. With a target sample of 14 for the laboratory sub-study, we can estimate feasibility parameters as follows: laboratory recruitment rate of 40% (14/35) with a 95% CI from 24 to 56% and post-BCT lab visit retention rate of 85% (12/14) with a 95% CI from 64 to 100%.

We used descriptive statistics to summarize recruitment, retention, data collection rates, and baseline characteristics. Statistical analyses were conducted using R version 4.1.1.

## Results

### Recruitment and enrollment

Recruitment was completed between December 2017 and February 2018. During this 3-month period, 105 soldiers were approached. Four were excluded: two did not meet inclusion criteria (< 18 years old, prior BCT exposure), and two declined to participate. A total of 101 people, including 31 females, were enrolled in the cohort (recruitment rate = 96.2%) and responded to the baseline assessments. Table 2 presents demographic and military characteristics of the enrolled participants. To evaluate the feasibility of recruiting participants into the laboratory sub-study, we targeted the first 24 participants who had ship dates later than 2 weeks post-enrollment and sent recruitment letters. Of these, 10 were excluded (4 did not respond, 6 were screened but declined or canceled lab visit appointment). A total of 14 participants were consented and completed pre-BCT lab visits, reflecting a laboratory recruitment rate of 58.3%.

**Table 2:** Baseline characteristics of the enrolled sample compared to follow-up survey responders and nonresponders

Baseline characteristic	Baseline sample (*N* = 101)		Follow-up survey responders (*n* = 73)		Follow-up survey nonresponders (*n* = 24)	
	*n*	%	*n*	%	*n*	%
Gender						
Male	70	69.3%	51	69.9%	16	66.6%
Female	31	30.7%	22	30.1%	8	33.3%
Age (years), mean (SD)	20.6 (3.5)		20.8 (3.7)		19.5 (1.9)	
18 years	29	28.7%	19	26.0%	9	37.5%
19 years	31	30.7%	23	31.5%	8	33.3%
20–24 years	28	27.7%	20	27.4%	6	25.0%
≥ 25 years	13	12.9%	11	15.1%	1	4.2%
Highest education level						
No postsecondary education	45	44.6%	28	38.4%	16	64.0%
Some postsecondary education	38	37.6%	29	39.7%	7	29.1%
Completed postsecondary degree	18	17.8%	16	21.9%	1	4.2%
Race						
White	76	75.2%	56	76.7%	18	75.0%
Non-white	25	24.8%	17	23.3%	6	25.0%
AFQT, mean (SD)	65.9 (22.6)		67.1 (23.3)		61.3 (21.1)	

Note: *AFQT* Armed Forces Qualification Test

### Retention

Figure 1 shows the number of participants who shipped to BCT and were followed up post-BCT. Among the 101 participants enrolled in the study, four participants did not ship to BCT during the study period (including 1 lab participant) and were ineligible for follow-up. A total of 97 participants were contacted after BCT, of which 73 responded to the follow-up survey (75.3% retention rate). Table 2 reports the baseline characteristics of participants who were retained in the longitudinal study (i.e., follow-up survey responders) compared to those who did not respond to the follow-up survey. The average time between the initial baseline data collection and follow-up data collection was 199 days (*SD* = 62). Following BCT, 13 of the 13 eligible lab participants completed post-BCT follow-up laboratory visits (100% retention rate). The average interval between pre-BCT and post-BCT lab visits was 170 days (*SD* = 56).

### Data collection and completeness

At baseline, 99 out of 101 participants provided complete data defined as responding to a minimum of 90% of the key variables (98.0% data completion rate); two participants were not able to complete the Penn CNB because of time restraints. For the follow-up assessment, 29 of the 73 responders (39.7% data completion rate) provided complete follow-up data when the Penn CNB was included in key variables used to calculate data completion rate. When we excluded the Penn CNB in key variables, 71 of the 73 participants (97.3% data completion rate) provided complete follow-up assessments. For the laboratory sub-study, 13 of 14 (92.9% lab completion rate) had complete lab visits at the pre-BCT time point. One participant was unable to complete the MRI because they met exclusion (profession as a machinist) following an additional safety screening. This was not initially discovered during screening for eligibility for the laboratory sub-study. At lab follow-up, the 13 participants eligible for follow-up completed all lab tasks, for a 100% lab completion rate.

### Exploratory outcomes

The nested RCT examined our ability to collect real-time stressor exposure data from participants in the study. Of the 50 participants randomly assigned to receive a brief web-based survey while at BCT, 1 completed the week 3 survey, 2 completed the week 6 survey, and 3 completed the week 9 survey. An average of 9.2 emails were sent to participants randomized to receive web-based surveys during BCT.

## Discussion

In this pilot study, we aimed to investigate the feasibility of recruiting and retaining young National Guard soldiers in preparation for a large-scale multilevel longitudinal cohort study focused on resilience in military populations. We met all preset progression criteria for testing the feasibility of the study methods and procedures to be used in the planned large-scale study. Our study demonstrates the feasibility of adapting existing resilience research models to incorporate pre-stressor baseline assessments within a more sophisticated design involving multiple levels of measurement. Importantly, we have successfully established the feasibility of a multilevel approach with an embedded laboratory sub-study.

One of our primary aims was to examine the feasibility of recruiting a unique population of young adults at the onset of their military careers. We succeeded in meeting pre-set recruitment goals of recruiting at least 100 participants for the main cohort and 14 participants for the laboratory sub-study cohort. We were concerned about the novel challenge of recruiting young soldiers as most existing literature focuses on deployment of soldiers later in their military careers. Because young soldiers may have less experience with the concept of resilience when they first enter the military, we thought it would be possible that new recruits would not be invested in a study researching the topic of resilience. However, our high recruitment rates achieved for the cohort study (96%) and laboratory sub-study (58%) demonstrate that our study engagement procedures were effective.

A second major aim was to examine the feasibility of retaining participants in the study after they returned from BCT. We were able to meet our goal of achieving retention rates over 65% for the main cohort and 85% in the laboratory sub-study cohort. Prior to starting, we had concerns about participant retention due to reassignment to various roles and units within the National Guard after BCT. Although our sample represented varying stages of young adulthood (e.g., some were in high school, and some were older), all recruits were juggling the dual responsibilities of the National Guard and civilian life. Initially, we encountered challenges with achieving acceptable follow-up survey retention rates. We found that initiating follow-up outreach efforts immediately after we anticipated soldiers would return from BCT was problematic and associated with lower-than-expected response rates. We learned that the post-return period was quite busy for recruits. Many participants were engaged in activities such as moving, starting new jobs, and reintegrating into school. Beyond affirming our concerns about the busy post-BCT transition period, we also learned that we lacked precise return dates. Ship dates frequently shifted slightly before BCT, with some participants sent to training early or late, and occasionally, training was extended longer than expected. Because there was no communication with participants planned for the period between study enrollment and BCT, we had no way of knowing if ship dates had shifted, which contributed to the variability of return dates. To address these challenges, we adjusted our participant outreach protocol for the main cohort by lengthening the time between anticipated BCT return date and start of follow-up efforts. After these modifications, we observed a positive trend in survey responses and were able to meet our retention rate feasibility aim. Because our procedures for the laboratory component involved calling lab participants to schedule post-BCT lab visits, we did not have the same difficulties with fluctuating return dates and were able to schedule and collect data for all participants.

Our third aim was to evaluate the completeness of data collected through surveys and laboratory visits from the participants we enrolled. We reached our goal of 90% data completeness survey data collected from the main cohort and 80% data completeness for the laboratory sub-study. However, we encountered challenges collecting Penn CNB data at follow-up when the measure was administered via personal devices. At baseline, participants were willing and able to complete the web-based survey and the Penn CNB within the time allotted. Only two participants were unable to complete the Penn CNB due to time constraints. At the post-BCT follow-up, we were able to achieve our completion milestone for the web-based survey but not the Penn CNB. Only 29 people completed the Penn CNB at follow-up. One possible reason was the length of the combined assessment [20]. Additionally, the Penn CNB required specific hardware and software specifications, which may have created barriers to response. For example, participants were able to complete the web-based survey on mobile devices, but the Penn CNB required a desktop computer or laptop. Moreover, the Penn CNB could be administered only on the Firefox browser with the Adobe Flash plug-in. The hardware/software requirements were difficult to troubleshoot remotely and made the Penn CNB inaccessible for many participants. Regarding completeness of laboratory data collected, participants completed all lab tasks at both time points apart from one lab participant who was excluded from completing the fMRI session due to safety issues.

The results of the RCT demonstrated that it was not feasible to collect self-reports of BCT stressor exposure during training. We encountered extremely low response rates to brief during-BCT surveys, and thus, we concluded that participants were not realistically available for study participation at this time. During BCT, recruits only had limited time, typically 30–60 min per week, to use personal electronic devices for leisure activities and communication with family. Responding to survey questions may not have been a priority for them. This insight has implications for studies involving similar military populations, including recruitment for personnel deployed in theater combat operations, where the success of survey administration may depend on survey designs that integrate well into soldiers’ actual availability during deployment [7].

### Recommendations

Although the results of our pilot study met all pre-determined progression criteria for determining whether the methods and procedures planned for the larger study were feasible, we identified several areas in our protocol that required modification to ensure the success of the large-scale study. The results of the RCT exploring the feasibility of collecting during-BCT surveys revealed that few soldiers responded during training, prompting us to abandon this approach and shift our focus towards outreach efforts targeting recruits upon their estimated return. Results examining the feasibility of collecting computerized neurocognitive data with the post-BCT follow-up survey also had low completion rates. Consequently, the larger study was modified to remove these procedures. Findings from this pilot study underscore the importance of designing assessment protocols to increase response rates and data quality in longitudinal studies. Additionally, we recognize the need for concise participant communication in future endeavors.

## Conclusion

The primary goal of the present study was to investigate the feasibility of recruiting and retaining young National Guard soldiers to participate in a prospective, longitudinal study of resilience with an embedded laboratory sub-study. Our approach to participant recruitment and retention proved feasible with this unique population of young adults at the beginning of their military careers. Moreover, our findings demonstrate that adapting previous research models of resilience to include pre-stressor baseline assessments and multiple levels of measurement was possible. However, several challenges were encountered, including low response rates during BCT and difficulties in completing neurocognitive tests on personal devices during follow-up. These challenges highlight the need for potential procedural changes in future iterations of the study. Furthermore, the present study emphasizes the importance of carefully designing assessment protocols to minimize barriers to response, particularly among populations with limited access to technology or subject to external factors that may affect their availability for study participation.

Based on our findings, we conclude that conducting a large-scale longitudinal cohort study on resilience among young National Guard recruits would be feasible with minimal changes to the study protocol. This research would significantly contribute to our understanding of resilience within this specific population.

## Data Availability

The datasets used and/or analyzed during the current study are available from the corresponding author upon reasonable request.
